# Cangfudaotan Decoction Alleviates Insulin Resistance and Improves Follicular Development in Rats with Polycystic Ovary Syndrome via IGF-1-PI3K/Akt-Bax/Bcl-2 Pathway

**DOI:** 10.1155/2020/8865647

**Published:** 2020-11-24

**Authors:** Chenye Wang, Caifei Ding, Zhoujia Hua, Chunyue Chen, Jia Yu

**Affiliations:** Department of Reproductive Medicine, Zhejiang Chinese Medicine and Western Medicine Integrated Hospital, Hangzhou, 310003 Zhejiang, China

## Abstract

Polycystic ovary syndrome (PCOS) is the most common endocrine and metabolic disorder prevalent in females of reproductive age; insulin resistance (IR) is the major pathogenic driver. Pharmacology is a basic option for PCOS therapy; traditional Chinese medicine (TCM), as a significant part of complementary and alternative medicine, has a long history in the clinical management of PCOS. Cangfudaotan decoction (CFD) has been used clinically for gynaecological diseases especially PCOS. In this study, first, chemical components in CFD were clarified using UPLC-Q/TOF-MS analysis. Then, an animal model of PCOS was established, granular cells were also isolated from the rats with PCOS, and CFD was administrated at different dosages in PCOS rats and granular cells, to investigate the therapeutic effect and mechanisms of CFD for PCOS treatment. The result showed that CFD treatment is effective in PCOS rats and granulosa cells. CFD was able to improve IR, restore the serum hormone levels, inhibit the inflammatory cytokines in PCOS rat, and alleviate ovary morphological injury and apoptosis in PCOS rats. In granulosa cells of PCOS, the result showed that the cell viability was improved, and cell apoptosis was inhibited after CFD administration. Further experiments suggested that CDF improves IR, follicular development, cell apoptosis, and inflammatory microenvironment, and this was associated to the regulation of IGF-1-PI3K/Akt-Bax/Bcl-2 pathway-mediated gene expression. Given that CFD sufficiently suppresses insulin resistance and improves follicular development in this study, exploring these mechanisms might help to optimize the therapeutic treatment of CFD in PCOS patients.

## 1. Introduction

Polycystic ovary syndrome (PCOS) is the most common endocrine and metabolic disorder prevalent in females of reproductive age. Usually, PCOS is characterized by hyperandrogenism, irregular menstrual cycle, abnormal ovarian function, follicular dysplasia (with multiple cystic ovarian follicles), and insulin resistance (IR) [1]. IR is the major pathogenic driver of PCOS; approximately 75% of PCOS patients have an impairment of insulin action [2]. In PCOS patients, ovarian cells are hyperresponsive to the stimulatory effects of insulin, thus causing ovarian hyperplasia; IR also amplifies hyperandrogenism, resulting in a disruption in the hypothalamic-pituitary-ovarian axis and aggravating PCOS [3]. Under the impairment of IR, abnormal apoptosis and dysregulation of granulosa cells are also observed [4]. In addition, systemic low-grade chronic inflammation also appears in women with PCOS, with some parameters at hypo- or hyperlevels [5]. Case-control studies show that PCOS is associated with lower life quality and increased psychological distress; both PCOS and thereby induced infertility have negative impact on reproductive age women [6, 7]. Moreover, in a female with PCOS, except for the great probability of infertility, the risks of endometrial carcinoma, diabetes mellitus, and cardiovascular diseases also seem to be higher than the normal populations [8, 9]. Hence, it is necessary for PCOS patients to be cured of reproductive disorders.

Pharmacology management is the major therapeutic option of PCOS patients. Metformin hydrochloride (MH) is commonly used as an insulin sensitizer to restore the elevated insulin and androgens levels and BMI; clomiphene citrate, developed as antiandrogens, is another way to resolve hyperandrogenism [10, 11]. Oral contraceptive pill (OCP) is focused on regularizing menstrual cycles and sex hormone levels [12]. Pharmacological therapies are useful to a certain extent, but we have to admit that these therapeutic medications are not totally effective and side effect-free. Long-term usage of these hormone manipulators can cause some side problems, including obesity, cancer, lactic acidosis, liver toxicity, ovarian hyperstimulation syndrome (OHSS), or miscarriage, even at low risk [13, 14]. Therefore, complementary treatments can be proper alternatives. Traditional Chinese medicine (TCM), as a significant part of complementary and alternative medicine, has a long history of usage in PCOS management. Various TCM herbs and prescriptions, which contain multiple active compounds without major adverse effects, are proposed in the treatment of clinical and laboratory symptoms of PCOS [15].

Cangfudaotan decoction (CFD), also known as Changbudodam-tang, is a TCM prescription which originates from a classic TCM book of gynecology. Several randomized controlled clinical trials show that CFD administration or combination with Western medications has the efficacy and safety on patients with PCOS [16, 17]. Although CFD has been used clinically for gynaecological diseases especially PCOS for a long time, its mechanism of action remains unclear because of its complex composition. A network pharmacology analysis that focuses on exploring the active ingredients and related pathways of CFD for treating PCOS shows that the PI3K-Akt, IR, Toll-like receptor, MAPK, and HIF-1 signaling pathways are related to the treatment of PCOS [18]. This preliminary analysis provides a possible clue for the pharmacodynamic mechanism research of CFD. In the present study, an animal model of PCOS was established, granular cells were also isolated from the rats with PCOS, and CFD was administrated at different dosages *in vivo* and *in vitro*, to investigate the therapeutic mechanisms of CFD for PCOS treatment.

## 2. Materials and Methods

### 2.1. Preparation and Identification of CFD

CFD was prepared by the Zhejiang Jingyuetang Pharmaceutical Co., Ltd (Shaoxing, China) from the same batch. CFD contains Rhizoma Atractylodis (Chinese pinyin name Cangzhu, 15 g), Rhizoma Cyperi (Chinese pinyin name Xiangfu, 10 g), Pinellia ternata (Chinese pinyin name Banxia, 9 g), Pericarpium Citri Reticulatae (Chinese pinyin name Chenpi, 6 g), Poria Cocos (Chinese pinyin name Fuling, 12 g), Arisaematis Rhizoma (Chinese pinyin name Tiannanxing, 6 g), Astragalus membranaceus (Chinese pinyin name Huangqi, 15 g), Fructus Aurantii (Chinese pinyin name Zhike, 10 g), Codonopsis Radix (Chinese pinyin name Dangshen, 15 g), Herba Epimrdii (Chinese pinyin name Yinyanghuo, 15 g), Crataegus pinnatifida Bge (Chinese pinyin name Shanzha, 30 g), Radix Salviae (Chinese pinyin name Danshen, 20 g), licorice (Chinese pinyin name Gancao, 5 g), white mustard (Chinese pinyin name Baijiezi, 10 g), Endothelium Corneum Gigeriae Galli (Chinese pinyin name Jineijin, 30 g), and Gleditsiae Spina (Chinese pinyin name Zaojiaoci, 10 g). In order to analyze the main phytochemicals in CFD, an UPLC-Q/TOF-MS technology was applied. UPLC-Q/TOF-MS analysis was performed on a Waters ACQUITY I-Class Plus UPLC system (Waters, USA) coupled to a SCIEX X-500R Q/TOF-MS (AB SCIEX, USA). The operation was conducted in both positive and negative ionization modes. The data was analyzed using SCIEX OS software, and the compounds were identified according to the MS data and matched to the TCM MS/MS Library.

### 2.2. Animals and PCOS Animal Models

The female SD rats (180 ± 20 g) were purchased from the Shanghai Sippr-BK Laboratory Animal Co. Ltd. (Certificate No. SCXK (Hu) 2018-0006; Shanghai, China) and housed in Zhejiang Chinese Medical University Animal Experiment Center (Certificate No. SYXK (Zhe) 2013-0184) in temperature 23 ± 2°C and humidity 60 ± 10% under 12 h light-dark cycles; water and food were available *ad libitum*. All animal experiments were approved by the Zhejiang Chinese Medical University Committee on Laboratory Animals, and the experiments were performed in strict accordance with the guidelines of the Chinese Council on Animal Care. After a week of adaptation to the laboratory conditions, rats were randomly divided into the control and experiment groups (*n* = 10 in each group). Control group rats were fed with normal diet during all the period of the experiments and intraperitoneally injected with 0.5% carboxymethyl cellulose (CMC, 1 ml/kg/d) for 21 days and then were orally administered with saline (1 ml/kg/d) for the next 4 weeks. For the experiment groups, rats were fed with high-fat diet (HFD) and intraperitoneally injected with 1.0 mg/kg of letrozole (1 ml/kg/d, dissolved in 0.5% CMC) for 21 days [19]. During this period, body weight and estrous cycle of rats were recorded; vaginal smears were performed to assess whether the PCOS model was successfully established. After the PCOS animal model was successfully established, the model rats were further randomly divided into the PCOS group (saline), the PCOS+L-CFD group (intragastrically administrated with CFD at a dosage of 15 g/kg/d), the PCOS+H-CFD group (intragastrically administrated with CFD at a dosage of 30 g/kg/d), and the PCOS+MH group (intragastrically administrated with MH at a dosage of 50 mg/kg/d). Animals were treated for 4 weeks.

### 2.3. Sample Collection and IR Calculation

After 12 h fasting after the last administration, all rats were weighed, and then, blood samples were obtained from the tail vein to assess fasting blood glucose (FBG) and fasting insulin (FINS) levels. HOMA-IR was calculated according to the following formula: IR = FBG (mmol/l) × FINS (m U/l)/22.5. Then, all rats were euthanized, blood samples were collected from the abdominal aorta, and serum samples were isolated by centrifugation at 3000 rpm for 15 min and then stored at -80°C for further experiments. The left and right ovaries were dissected and weighed; the diameter of both ovaries was also detected.

### 2.4. ELISA

Serum follicle-stimulating hormone (FSH), luteinizing hormone (LH), testosterone (T), estradiol (E2), TNF-*α*, IL-1*β*, IL-6, and CRP levels were determined using commercially available ELISA kits (MEIMIAN, China). All procedures were performed according to the manufacturer's instructions. All samples were analyzed in duplicate.

### 2.5. Histological Analysis

Then, ovarian tissues were fixed in 4% paraformaldehyde, embedded in paraffin, sectioned at 4 *μ*m thickness, and stained with hematoxylin and eosin (H&E). Then, sections were viewed and photographed using a Leica DM3000 microscope (Leica, Germany). TUNEL staining was performed to assess the cell apoptosis in ovarian tissues. Briefly, ovarian tissue sections were incubated with the TUNEL reaction mixture in a humidified chamber at 37°C for 1 h (Beyotime Biotechnology Co., Ltd., Shanghai) and further incubated with peroxidase-conjugated HRP antibody at 37°C for 30 min. Then, immunoreactivity was visualized using 3,3′-diaminobenzidine (DBA), and the TUNEL-positive cells in ovarian tissue sections were observed and photographed using a Leica DM3000 microscope (Leica, Germany).

### 2.6. Immunohistochemical (IHC) Analysis

IHC staining was performed to assess the expression of Bax and Bcl-2 in ovarian tissues. First, ovarian tissue sections were subjected to 0.1 M citrate buffer (pH 6.0) at 95~100°C for 1 h. After blocking with 3% BSA, sections was incubated with primary antibody (Bax: 1 : 200 dilution, ab32503, Abcam, USA; Bcl-2: 1 : 200 dilution, ab182858, Abcam, USA) at 4°C overnight. After washing with PBS for three times, the section was further incubated with horseradish peroxidase- (HRP-) conjugated secondary antibody for 20 min at room temperature. Then, immunoreactivity was visualized using DBA and examined using a Leica DM3000 microscope (Leica, Germany).

### 2.7. Preparation of CFD-Containing Serum

Female SD rats were intragastrically administrated with normal saline (control) or CFD (15 g/kg/d and 30 g/kg/d) once daily for seven consecutive days. Two hours after the last administration, blood samples were collected from the aorta ventralis of each group's rats, saved at 4°C for 1 h, and then centrifuged at 3000 rpm/min for 15 min. The obtained blood samples from the same group were mixed together, inactivated by incubating in a water bath at 56°C for 30 min for, and sterilized through microporous membrane filtration. Then, the CFD-containing serum samples were stored at -80°C for subsequent *in vitro* experiments.

### 2.8. Granular Cell Isolation and Identification

Granular cell isolation was conducted according to the protocol previously described [20]. Briefly, normal and PCOS rat ovaries were removed and placed in serum-free high-glucose DMEM; adipose and connective tissues were removed. Then, the ovaries were washed and suspended in DMEM/F12. Then, follicle puncture was performed with fine needles under microscopic visualization, and granular cells were released into the medium. Then, cells were filtered through 200 *μ*m nylon meshes, allowing granular cells to pass through. The collected granular cells were centrifuged, and the supernatant was thereupon cultured in fresh 10% FBS-DMEM/F12 at 37°C in a 5% CO_2_ atmosphere. Cell morphology was observed under a Ts2-FC inverted fluorescence microscope (Nikon, Japan); HE assay was performed to identify the isolated granular cells.

### 2.9. Cell Treatment and CCK-8 Cell Viability Assay

Cell viability was detected with CCK-8 assay. Briefly, granular cells were seeded in 96-well plates for 24 h and further treated with CFD (CFD-containing serum, 0.1 mg/ml, 0.2 mg/ml), MK-2206 (PI3K inhibitor, 20 *μ*M), LY294002 (Akt inhibitor, 20 *μ*M), or 20 *μ*M IGF-1 for 12 h, 24 h, 48 h, and 72 h, respectively. Then, 10 *μ*l of CCK-8 solution was added and further incubated at 37°C for 1.5 h. The optical density (OD) of each was detected 490 nm with a CMax Plus microplate reader (MD, USA). The cell viability with different treatment conditions was then calculated from their corresponding OD values.

### 2.10. Flow Cytometry Analysis

Cell apoptosis was assessed using the Annexin V/fluorescein isothiocyanate (FITC)/propidium iodide (PI) apoptosis detection kit (BD Pharmingen, USA). Generally, granular cells in logarithmic phase were seeded in 6-well plates at a density of 1.2 × 10^6^. Then, cells were washed by binding buffer, centrifuged, and resuspended in 100 *μ*l binding buffer. 5 *μ*l Annexin V-FITC and 10 *μ*l PI solution were added to the suspension under dark room for 15 min. 400 *μ*l binding buffer was added, and cell apoptosis was analyzed using a FC500 flow cytometer (Beckman, USA). The percentage of early and late phase of apoptotic cells was calculated as cell apoptotic rate (%).

### 2.11. Western Blotting Assay

Granular cells from each group were homogenized in ice-cold RIPA lysis buffer (Beyotime Biotechnology Co., Ltd., China) for 30 min, and the supernatants were isolated after centrifugation at 12,000 g for 5 min at 4°C. The total protein concentration was determined using BCA kit (Solarbio, China). After that, 50 *μ*g of protein samples was separated on 10% SDS-PAGE gels in Tris-glycine and 0.1% SDS buffer and transferred to PVDF membranes. After blocking with 5% nonfat milk and washing with TBST for three times, the membranes were incubated with the primary antibodies (BAD, Bax, and Bcl-2: CST, USA; IGF-1, Akt, p-Akt, and GAPDH: Abcam, UK; PI3K, p-PI3K: Affinity Biosciences, USA) at 4°C overnight. Then, after blocking with 5% nonfat milk and washing with TBST for three times, the membranes were further incubated with the HRP-conjugated goat anti-rabbit IgG secondary antibody (Abcam, UK) for 1 h at room temperature. The protein bands were detected using ECL reagents and visualized by a chemiluminescence capture system. The quantification was carried out using ImageJ software, and GAPDH was used as the internal control.

### 2.12. Statistical Analysis

The data are presented as mean ± SD. Differences between the two groups were analyzed using the Student *t* test; differences among more than two groups were analyzed by conducting one-way ANOVA followed by LSD comparison test using SPSS 18.0 software (SPSS, USA). *P* < 0.05 was considered as statistically significant.

## 3. Results and Discussion

### 3.1. Determination of Chemical Components in CFD

**Figure 1:**
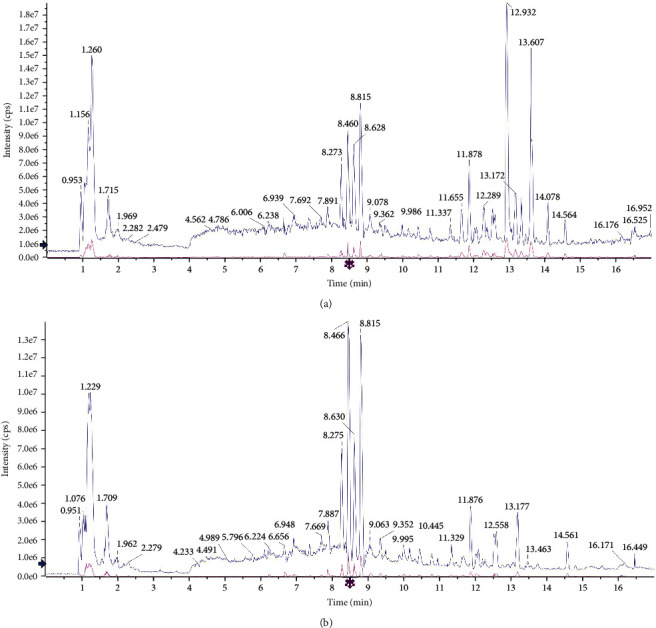
Characteristics of chemical components in CFD by UHPLC-Q-TOF/MS analysis in the (a) positive ion mode and (b) negative ion mode.

Characteristics of chemical components in CFD were analyzed by UHPLC-Q/TOF-MS under positive ion mode and negative ion mode. As shown in Figure 1 and Table 1, UHPLC-Q-TOF/MS analysis in positive ion mode identified 43 compounds in CFD, including betaine, stachydrine, narirutin, hesperetin, and nobiletin. UHPLC-Q/TOF-MS analysis in negative ion mode also identified 43 compounds in CFD; some of them were the same with the compounds in the positive mode.

### 3.2. Changes of Estrous Cycle in PCOS Rat Models

**Figure 2:**
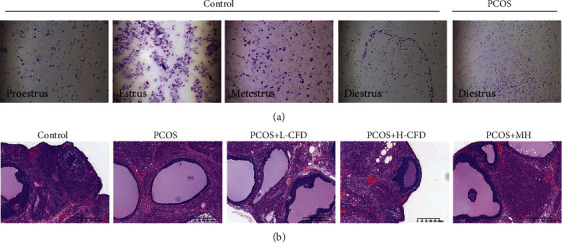
Effect of CFD on estrous cycle and ovary morphological alterations of PCOS rat model: (a) changes of estrous cycle in rats; (b) H&E staining (magnification ×200).

The vaginal smears of the rats in the normal control and PCOS groups are presented in Figure 2(a). Rats in controls had normal 4-5 days estrous cycles, comprising proestrus, estrus, metestrus, and diestrus. Nucleated epithelial cells were observed in proestrus; keratinocytes with irregular shape that interconnected into pieces were observed in estrus; nucleated epithelial cells, irregular epithelial keratinocytes, and leukocytes were observed in metestrus; and a large number of leukocytes were observed in diestrus. But with letrozole administration, there was an irregular estrous cycle in PCOS group rats, with prolonged diestrus phase, and a large number of leukocytes were observed.

### 3.3. Effect of CFD on Ovary Weights and Diameter and Ovary Index of PCOS Rat Model

At the end of the experiments, rat body weight and left and right ovary weight and diameter were detected. It could be observed that with HFD feeding, the body weight was significantly increased in PCOS rats. As shown in Table 2, compared to the control group, rat ovary diameter and organ index in the PCOS group were significantly increased (*P* < 0.01). Compared to the PCOS model group, in the CFD low-dose group, the ovary index was significantly decreased (*P* < 0.05); in the CFD high-dose group, the ovary diameter and ovary index were significantly decreased (*P* < 0.05).

### 3.4. Effect of CFD on Ovary Morphological Recovery of PCOS Rat Model

H&E staining (Figure 2(b)) showed that the structure in the control group ovarian tissues exhibited a normal morphology, with multiple luteal and follicles, as well as normal oocytes, and multiple layers of granular cells within the follicles were observed. But the ovary in the PCOS group showed multiple follicles with apparent cystic dilatation, and the layers of the granular cells were decreased, and the oocytes within the follicles also seemed to disappear. Upon treatment with CFD, it showed that the morphological alterations in PCOS rat ovary were recovered partly, the oocytes appeared, the layers of the granular cells increased, and the cystic follicles also decreased.

### 3.5. Effect of CFD on IR, Serum Hormone, and Proinflammatory Cytokine Levels of PCOS Rat Model

**Figure 3:**
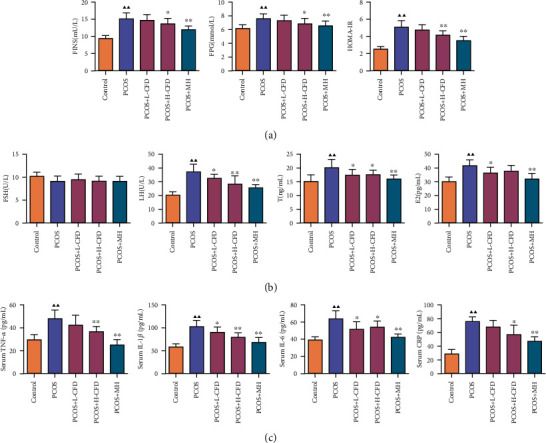
Effect of CFD on PCOS rat (a) insulin resistance, (b) serum hormone levels, and (c) proinflammatory cytokine levels (*n* = 10). Compared to the control group, ^▲^*P* < 0.05 and ^▲▲^*P* < 0.01; compared to the PCOS group, ^★^*P* < 0.05 and ^★★^*P* < 0.01.

The FPG and FNS levels were detected; the HOMA-IR was also calculated in each group's rats. It could be observed that the levels of FPG and FNS and HOMA-IR were increased in PCOS model rats, but these increases were restored with CFD or MH treatment, especially in the H-CFD and MH groups with significant differences compared with the PCOS group (*P* < 0.05, *P* < 0.01; Figure 3(a)). Serum hormone levels including FSH, LH, P, and T were also detected to assess the effect of CFD on serum hormone (Figure 3(b)). The results showed that there are no significant changes of the FSH levels among the groups, while LH, T, and E2 levels in the PCOS group were significantly increased compared with those in the control group (*P* < 0.01). Compared with the PCOS model group, the levels of LH and T were significantly decreased in the CFD-treated groups (*P* < 0.05). As shown in Figure 3(c), serum levels of proinflammatory cytokines, including TNF-*α*, IL-1*β*, IL-6, and CRP, were also detected. In the PCOS group, the levels of TNF-*α*, IL-1*β*, IL-6, and CRP were significantly elevated compared with those in the control group, but these elevated levels were significantly inhibited in the CFD treatment groups (*P* < 0.05).

### 3.6. Effect of CFD on Cell Apoptosis and Bax and Bcl-2 Expression in Ovarian Tissues of PCOS Rat Model

**Figure 4:**
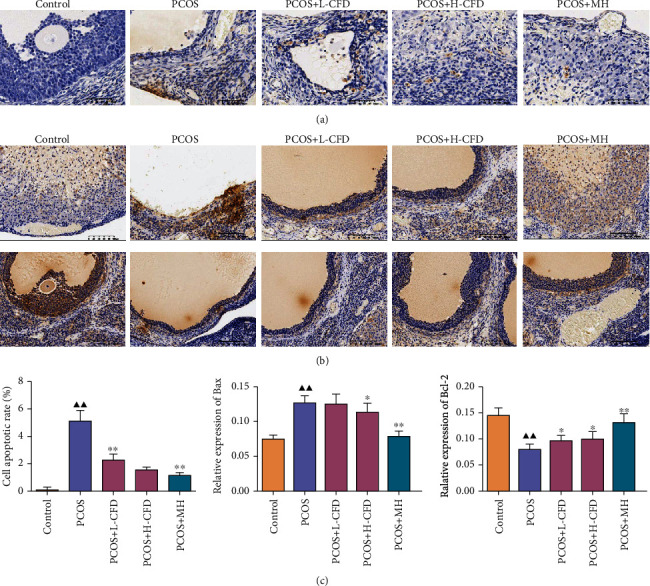
Effect of CFD on cell apoptosis and Bax and Bcl-2 expression in PCOS rat ovarian tissues (*n* = 3). (a) TUNEL staining; (b) immunohistochemistry staining of Bax and Bcl-2; (c) semiquantification of cell apoptosis and relative expression of Bax and Bcl-2. Magnification ×200.

TUNEL staining was used to assess the effect of CFD on cell apoptosis in ovarian tissues (Figure 4(a)). The result showed that the PCOS group exhibited a significant high rate of cell apoptosis compared with the control group. CFD treatment had an inhibition effect on the cell apoptosis in PCOS rat ovarian tissues, especially in high dosage. Since the TUNEL staining showed that CFD could inhibit cell apoptosis in PCOS model rats, IHC staining was further performed to determine the expression of Bax and Bcl-2 in ovarian tissues. As the results indicated in Figure 4(b), the relative expression of Bax was significantly increased; Bcl-2 was significantly decreased in the PCOS model group compared to the control group, while in CFD-treated groups the relative expression of Bax was decreased, and Bcl-2 was increased, with statistical significance in the L-CFD group for Bax and in the H-CFD group for Bax and Bcl-2 (*P* < 0.05).

### 3.7. Identification of the Isolated Granular Cells

**Figure 5:**
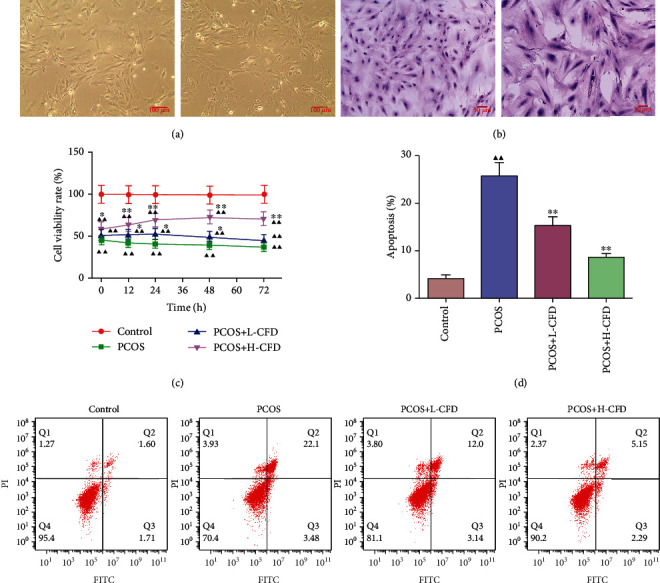
CFD ameliorates cell viability, inhibiting cell apoptosis in granular cells with PCOS (*n* = 3). (a) Cell morphology identification of the normal and PCOS model granular cells (magnification ×100); (b) H&E staining of the normal and PCOS model granular cells (magnification ×200); (c) granular cells were treated with CFD (0.1 mg/ml, 0.2 mg/ml) for 12 h, 24 h, 48 h, and 72 h, and then, CCK-8 assay was used to assess cell viability; (d) cell apoptosis was assessed using Annexin V/FITC/PI flow cytometry analysis.

Normal and PCOS model granular cells were isolated from the control and PCOS rat ovary, respectively. It could be observed that the granular cells in the control group had a pleomorphic or fusiform shape, and the cells were connected together by their pseudopodia; the cells in the PCOS group had a pleomorphic or fiber shape (Figure 5(a)). H&E staining showed that the granular cells in the control group had dark blue-stained cell nuclei, reddish-stained cytoplasm, and nucleolus were easy observed; the granular cell nuclei in the PCOS group were dark blue in stained color, oval in shape, with some cell nuclei enlarged (Figure 5(b)).

### 3.8. Effect of CFD on Cell Viability and Apoptosis in Granular Cells of PCOS Rat Model

CCK-8 assay was used to analyze the granular cell viability with or without CFD treatment for 0 h, 12 h, 24 h, 48 h, and 72 h. As the result indicated, the cell viability rate in the PCOS group was significantly inhibited compared to that in the control group in 0 h, 12 h, 24 h, 48 h, and 72 h (*P* < 0.01; Figure 5(c)). After incubation with CFD at different dosages, the results showed that the cell viability rates were increased compared with those of the PCOS group. In 24 h and 48 h, statistical differences were observed between the CFD groups and the PCOS group. Cell apoptosis was also evaluated using the flow cytometry assay (Figure 5(d)). After incubation with CFD for 24 h, it showed that the cell apoptosis rates were significantly increased in the PCOS group granular cells (*P* < 0.01), but upon CFD treatment, the apoptotic rate was decreased significantly (*P* < 0.01).

### 3.9. Effect of CFD on IGF-1-PI3K/Akt-Bax/Bcl-2 Pathway in Granular Cells of PCOS

**Figure 6:**
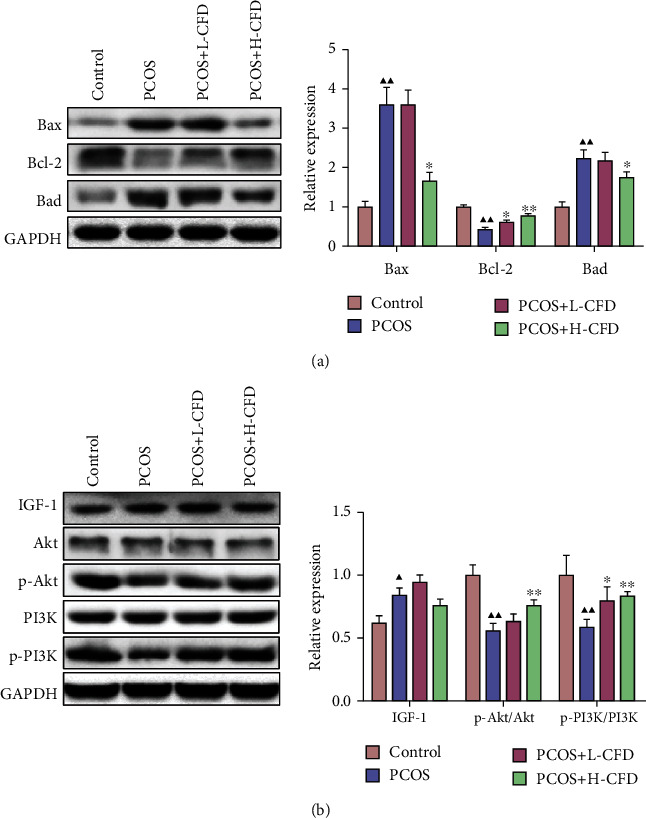
CFD ameliorates follicular development and insulin resistance via the IGF-1-PI3K/Akt-Bax/Bcl-2 pathway (*n* = 3). (a) Expression of Bax, Bcl-2, and Bad was measured by western blotting. (b) Expression of IGF-1, p-PI3K/PI3K, and p-Akt/Akt was measured by western blotting. GAPDH was used as control.

As shown in Figure 6(a), the relative protein expression levels of Bax and Bad were significantly increased, and Bcl-2 was significantly decreased in the PCOS model granular cells compared to the control granular cells (*P* < 0.01). Upon CFD administration, the expression level of Bcl-2 was significantly increased in the L-CFD group (*P* < 0.05), the expression levels of Bax and Bad were significantly decreased, and Bcl-2 was significantly increased in the H-CFD group (*P* < 0.05). As shown in Figure 6(b), the relative expression levels of IGF-1 were significantly increased, and p-Akt/Akt and p-PI3K/PI3K were significantly decreased in the PCOS model cells (*P* < 0.05). Upon CFD administration, the expression levels of p-Akt/Akt and p-PI3K/PI3K were significantly increased in the CFD groups (*P* < 0.05).

### 3.10. IGF-1-PI3K/Akt-Bax/Bcl-2 Pathway Involved in the Attenuation of CFD on PCOS Model Granular Cells

**Figure 7:**
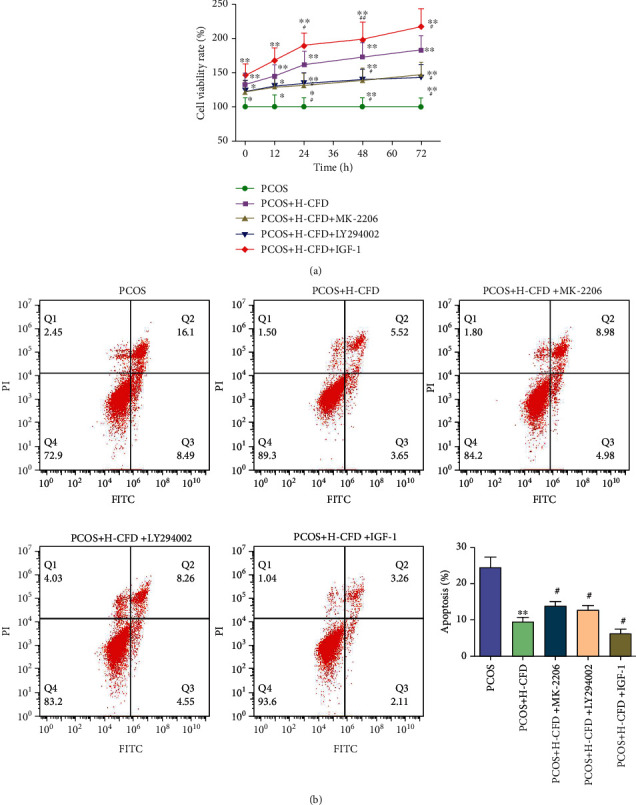
CFD ameliorates cell viability, inhibiting cell apoptosis in granular cells of PCOS rat model (*n* = 3). (a) Granular cells were treated with CFD (0.2 mg/ml), MK-2206 (20 *μ*M), LY294002 (20 *μ*M), or IGF-1 (20 *μ*M) for 12 h, 24 h, 48 h, and 72 h, and then, CCK-8 assay was used to assess cell viability. (b) Cell apoptosis was assessed using Annexin V/FITC/PI flow cytometry analysis.

To explore the role of the IGF-1-PI3K/Akt-Bax/Bcl-2 pathway in the attenuation of CFD on PCOS, PCOS model granular cells were incubated with CFD (0.2 mg/ml), together with 20 *μ*M MK-2206 (PI3K inhibitor), 20 *μ*M LY294002 (Akt inhibitor), or 20 *μ*M IGF-1. CCK-8 assay showed that compared to the PCOS group cells, the cell viability rates were significantly increased in the treatment groups (*P* < 0.01; Figure 7(a)). Furthermore, compared with the H-CFD group, the cell viability was significantly increased in the H-CFD+IGF-1 group; the cell viability was significantly increased in the H-CFD+MK-2206 group and the H-CFD+LY294002 group after 24 h, 48 h, and 72 h treatment (*P* < 0.05). Cell apoptosis results also showed that the apoptosis rates in the treatment groups were significantly decreased compared to those in the PCOS group (*P* < 0.01; Figure 7(b)). Compared to the H-CFD group, cell apoptosis was significantly increased in the H-CFD+MK-2206 group and the H-CFD+LY294002 group, and it was significantly increased in the H-CFD+IGF-1 group (*P* < 0.05).

**Figure 8:**
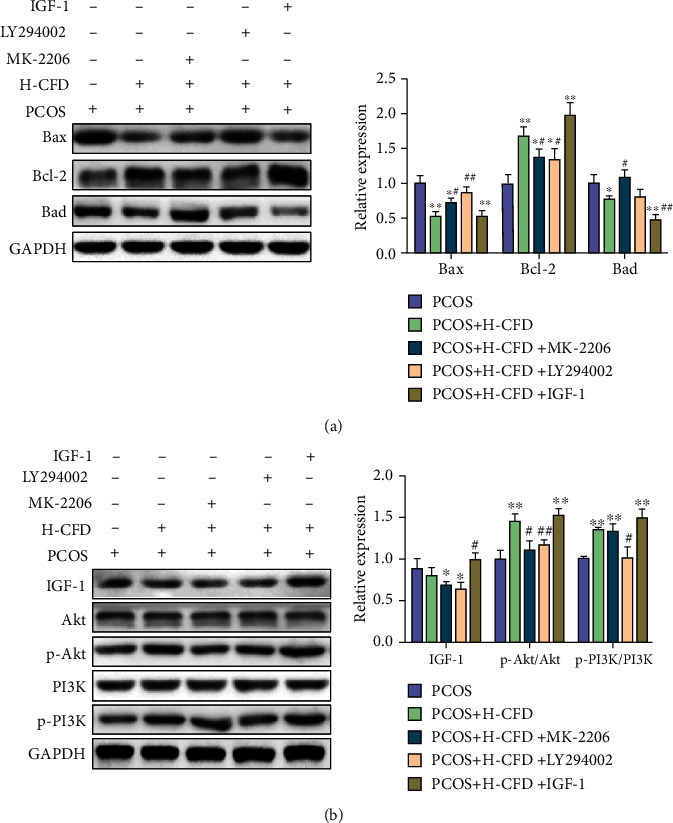
CFD ameliorates follicular development and insulin resistance via the regulation of the IGF-1-PI3K/Akt-Bax/Bcl-2 pathway (*n* = 3). (a) The expression of Bax, Bcl-2, and Bad was measured by western blotting. (b) The expression of IGF-1, p-PI3K/PI3K, and p-Akt/Akt was measured by western blotting. GAPDH was used as control.

As shown in Figure 8(a), the relative protein expression levels of Bax and Bad were significantly decreased, and Bcl-2 was significantly increased in the H-CFD granular cells compared to the PCOS model granular cells (*P* < 0.01). Compared to the H-CFD group, the expression levels of Bax and Bad were increased, and Bcl-2 was decreased in the H-CFD+MK-2206 group and the H-CFD+LY294002 group (*P* < 0.05); the expression level of Bad was significantly decreased in the H-CFD+IGF-1 group (*P* < 0.05). As shown in Figure 8(b), compared to the H-CFD group, the relative expression levels of IGF-1 were decreased; p-Akt/Akt and p-PI3K/PI3K were decreased in the H-CFD+MK-2206 group and the H-CFD+LY294002 group cells (*P* < 0.05). The expression levels of IGF-1, p-Akt/Akt, and p-PI3K/PI3K were increased in the H-CFD+IGF-1 group.

## 4. Discussion

Given to the conditions that PCOS continues to increase and has a negative impact on reproductive age females, it is necessary for PCOS patients to be cured of reproductive disorders. Based on a PCOS model in rat and granulosa cells, in this study, we found that CFD treatment for a period is effective *in vivo* and *in vitro*. It showed that CFD is able to improve IR, restore the serum hormone levels in PCOS rats, and alleviate ovary morphological injury and apoptosis in PCOS rats. In granulosa cells of PCOS, the result showed that the cell viability was improved, and cell apoptosis was inhibited after CFD administration. Further experiments suggested that CFD alleviated PCOS which contributed to the regulation of the IGF-1-PI3K/Akt-Bax/Bcl-2 pathway. In addition, characteristics of the chemical components in CFD were clarified using UPLC-Q/TOF-MS analysis; 43 compounds were identified under positive ion mode and negative ion mode.

The pathogenesis of PCOS remains complicated and not clear yet. But more and more studies evidence that IR regulates multiple mediators and pathways and is critically involved in the pathogenesis and development of PCOS [2, 21]. Insulin is the key regulatory hormone in the processes of glucose and lipid energy metabolism. In the present study, except for the significantly increased HOMA-IR in PCOS rats, the serum level of T and LH/FSH ratio were also significantly increased compared to those in normal rats, which corresponded with the previous studies. The abnormal increase of insulin could stimulate the oversecretion of androgen, thus inducing hyperandrogenism. A recent study published in “Lancet” demonstrated that insulin could drive adipose androgen generation in PCOS female subcutaneous adipose tissue, and this improvement is worked by increasing AKR1C3 activity [22]. Furthermore, an irregular estrous cycle, with a prolonged diestrus phase in PCOS rats, was observed via vaginal smears in this study. At the same time, HE staining showed that the ovary morphology in PCOS rats was different from the controls, characterized by multiple follicles with apparent cystic dilatation and the decrease of the granular cell layers and oocytes within the follicles. The excessive androgen in PCOS can cause early luteinization of ovarian granular layer cells, stop follicular development and growth, and lead to follicle atresia and eventually anovulation or poor ovulation [19]. Thereby, irregular menstrual cycle or estrous cycle also occurred. After CFD treatment, our result found that HOMA-IR was decreased. It seems that CFD could regulate the FPG and FINS levels to inhibit IR in PCOS rats, which was consistent with the clinical study of CFD [17]. In CFD-treated groups, the serum LH and T levels were restored, the injury on ovary morphology was recovered, and cell apoptosis was also inhibited, especially in the high-dosage group.

Chronic inflammation is accompanied with the occurrence and progression of PCOS. Continuous release of inflammatory mediators could perpetuate the inflammatory condition in women with PCOS, possibly associated with IR and other long-term clinical manifestations [23, 24]. Multiple mediators of inflammation, such as TNF-*α*, IL-1, IL-6, IL-8, CCL2, CRP, and MCP, as well as inflammatory-immune cells and increased oxidative stress contributed to the low-grade systemic inflammation of PCOS. A prospective case-control study shows that the serum TGF-*β*1 and NF-*κ*B were significantly higher and TSP-1 was significantly lower in the PCOS groups than those in the control group, which support the association between PCOS and chronic inflammation [5]. Hence, strategies focused on ameliorating inflammation may be possible management of PCOS [25, 26]. In our study, we detected the inflammatory cytokines, including TNF-*α*, IL-1*β*, IL-6, and CRP in PCOS; the result showed that the elevated serum levels of TNF-*α*, IL-1, IL-6, and CRP in PCOS model rats were significantly decreased with CFD treatment.

As a guarder of ovum, granulosa cells produce steroidal hormones and growth factors, interact with the oocyte, create a highly specialized microenvironment, and play an essential role in normal follicular development and maturation process [27]. Follicular dysplasia is a basic characteristic of PCOS, and accumulation studies show that the follicular dysplasia in PCOS is critically associated with the abnormal apoptosis of granulosa cells [20, 28]. The Bcl-2 family has a key role in the mitochondrial apoptosis pathway; the abnormal expression of proteins in the Bcl-2 family, such as Bcl-2, Bcl-XL, Bax, and Bad, is involved in the abnormal increased apoptosis in PCOS [29, 30]. In our study, TUNEL staining and flow cytometry analysis showed that cell apoptosis was significantly increased in PCOS rat ovary tissues and granulosa cells. In addition, it seemed that there is abnormal expression of Bcl-2 family proteins in PCOS, the expressions of Bax and Bad were increased, and Bcl-2 was decreased compared to the normal controls. But in the CFD treatment groups, inhibition effects on cell apoptosis *in vivo* and *in vitro* were observed, and the protein expression of the Bcl-2 family was also restored. It indicated that CFD might improve follicular development in PCOS rats by regulating apoptosis.

PI3K/Akt signaling is one of the classic insulin signaling pathways; insulin mainly regulates the PI3K/Akt signaling to mediate its metabolic regulation effect. Insulin receptor (INSR), as a receptor of insulin, usually combines with insulin receptor substrate (IRS) to activate two signaling pathways: PI3K and MAPK. The PI3K/Akt pathway could be induced via insulin and acts as an important effector; Akt/PKB is capable of translocating glucose transporters, like GLUT4, to the cell membrane thus increasing glucose uptake [31]. Cho et al.'s study published in “Science” showed that mice deficient in AKT2 are impaired in the ability of insulin to lower blood glucose [32]. Zhang et al. showed that in PCOS rats, the therapy effect of berberine in reducing PCOS pathology and IR values is associated with a mechanism linked to GLUT4 upregulation via PI3K/Akt activation and MAPK pathway suppression [33]. The aforementioned network pharmacology of CFD shows that CFD could regulate the IR and PI3K/Akt pathway for PCOS treatment, which suggested that the therapy effect of CFD in PCOS was related to the improvement of IR and might be via the PI3K/Akt pathway [18]. In this study, in PCOS rats with IR, the inhibition on PI3K/Akt signaling was also observed using western blotting. And CFD could restore this inhibition effect, which was enhanced with IGF-1 (PI3K activator) cotreatment but was counteracted with MK-2206 (PI3K inhibitor) or LY294002 (Akt inhibitor) cotreatment. Based on this, we suggested that CFD ameliorates ovary function and IR via the IGF-1-PI3K/Akt pathway. PI3K/Akt signaling is not only critically associated with IR but also involved in the regulation of cell survival, autophagy, and inflammatory microenvironment. Zhao et al. show that regulation of PI3K/Akt signaling is associated to the inflammation and oxidative stress in granulosa cells of PCOS patients [34]. In this study, the improvement on inflammatory microenvironment with CFD administration indicated that CFD might regulate PI3K/Akt signaling to suppress the production of proinflammatory cytokines, thus improving the inflammatory microenvironment in PCOS rats.

## 5. Conclusions

**Figure 9:**
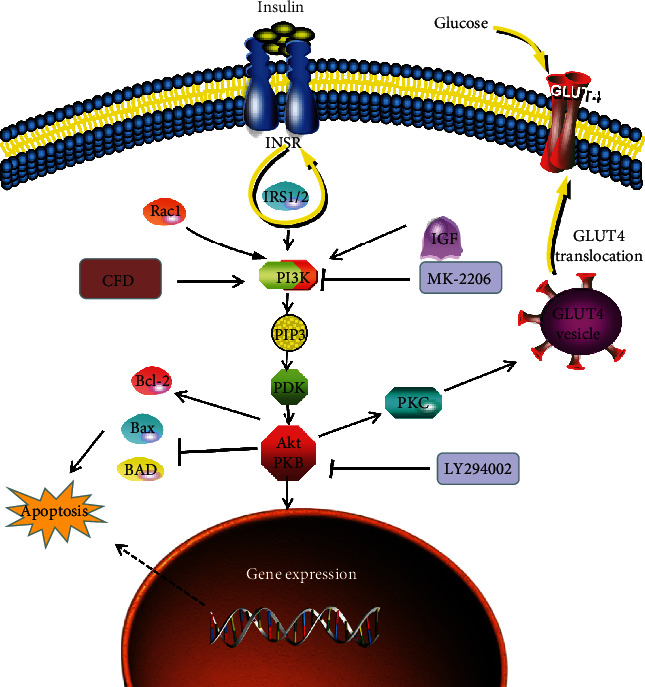
CFD ameliorates insulin resistance and improves follicular development in rats with polycystic ovary syndrome via the IGF-1-PI3K/Akt-Bax/Bcl-2 pathway.

In conclusion, based on a PCOS model, we found that CFD treatment had a therapeutic effect in PCOS rats and granulosa cells. It showed that CFD is able to improve IR, restore the serum hormone levels, inhibit the inflammatory cytokines, and alleviate ovary morphological injury and apoptosis in PCOS rats. In granulosa cells with PCOS, the result showed that cell viability was improved, and cell apoptosis was inhibited after CFD administration. Further experiments suggested that CDF improves follicular development and IR, inhibits apoptosis and inflammatory microenvironment, and contributes to the regulation of the IGF-1-PI3K/Akt-Bax/Bcl-2 pathway (Figure 9). Given that CFD sufficiently suppresses IR and improves follicular development, exploring these mechanisms might help to optimize the therapeutic treatment of CFD in PCOS patients.

## Figures and Tables

**Table 1:** Characteristics of chemical components in CFD by UHPLC-Q-TOF/MS analysis.

No.	Component name	Retention time	Formula	Precursor mass	Found at mass	Mass error (ppm)	Library score	Isotope ratio difference
1	L(+)-Arginine	1.08	C_6_H_14_N_4_O_2_	175.119	175.1191	0.7	89.8	1.0
2	Betaine	1.16	C_5_H_11_NO_2_	118.086	118.0865	2.0	100.0	0.4
3	Trigonelline	1.20	C_7_H_7_NO_2_	138.055	138.0552	2.1	95.8	0.2
4	Proline	1.23	C_5_H_9_NO_2_	116.071	116.0709	2.3	97.8	0.6
5	Stachydrine	1.26	C_7_H_13_NO_2_	144.102	144.1019	-0.3	96.4	1.3
6	Nicotinic acid	1.73	C_6_H_5_NO_2_	124.039	124.0396	2.4	99.5	0.1
7	Adenosine	3.68	C_10_H_13_N_5_O_4_	268.104	268.1044	1.4	100.0	1.8
8	Guanosine	4.12	C_10_H_13_N_5_O_5_	284.099	284.0995	2.0	100.0	2.2
9	Phenprobamate	4.78	C_9_H_11_NO_2_	166.086	166.0865	1.3	95.7	0.8
10	Esculin hydrate	5.88	C_15_H_16_O_9_	341.087	341.0881	4.0	92.4	2.8
11	Chlorogenic acid	6.24	C_16_H_18_O_9_	355.102	355.1027	0.9	99.5	0.4
12	Fraxetin	7.25	C_10_H_8_O_5_	209.044	209.0449	2.3	75.6	0.8
13	Eriodictyol	7.88	C_15_H_12_O_6_	289.071	289.0713	2.1	84.4	1.7
14	Polygalaxanthone IV	7.88	C_27_H_32_O_15_	597.181	597.1823	1.4	74.6	3.8
15	Liquiritigenin	7.94	C_15_H_12_O_4_	257.081	257.0814	2.3	95.3	4.3
16	Scopoletin	8.07	C_10_H_8_O_4_	193.050	193.0500	2.6	91.4	2.5
17	Isoferulic acid	8.09	C_10_H_10_O_4_	195.065	195.0657	2.6	97.0	1.1
18	Isorhamnetin	8.17	C_16_H_12_O_7_	317.066	317.0664	2.6	70.4	2.9
19	Narirutin	8.47	C_27_H_32_O_14_	581.186	581.1872	1.2	97.6	0.9
20	Naringenin	8.48	C_15_H_12_O_5_	273.076	273.0762	1.6	100.0	0.2
21	Hesperetin	8.82	C_16_H_14_O_6_	303.086	303.0865	0.6	99.4	0.9
22	Hesperidin	8.82	C_28_H_34_O_15_	611.197	611.1975	0.7	84.1	1.8
23	Apigenin 7-O-beta-D-glucuronide	9.37	C_21_H_18_O_11_	447.092	447.0928	1.4	100.0	2.6
24	6,7-Dimethoxycoumarin	9.40	C_11_H_10_O_4_	207.065	207.0656	2.2	96.5	1.5
25	Xanthotoxol	10.13	C_11_H_6_O_4_	203.034	203.0344	2.4	74.6	1.5
26	Wogonin 7-O-glucuronide	10.45	C_22_H_20_O_11_	461.108	461.1087	1.9	100.0	1.0
27	Glycocholic acid	11.34	C_26_H_43_NO_6_	466.316	466.3168	1.1	79.8	2.7
28	Sodium glycocholate	11.34	C_26_H_42_NNaO_6_	488.298	488.2987	0.9	97.0	3.3
29	Baicalein	11.64	C_15_H_10_O_5_	271.060	271.0604	1.1	89.1	0.8
30	Glycyrrhetinic acid	12.04	C_30_H_46_O_4_	471.347	471.3474	1.0	83.7	2.8
31	Formononetin	12.06	C_16_H_12_O_4_	269.081	269.0812	1.4	91.2	1.1
32	Bergapten	12.10	C_12_H_8_O_4_	217.050	217.0500	2.1	96.3	3.6
33	Limonin	12.54	C_26_H_30_O_8_	471.201	471.2020	1.4	97.0	0.4
34	Patchouli alcohol	12.61	C_15_H_24_	205.195	205.1955	2.0	85.5	3.9
35	Wogonin	12.90	C_16_H_12_O_5_	285.076	285.0762	1.7	93.6	2.1
36	Nobiletin	12.93	C_21_H_22_O_8_	403.139	403.1387	-0.2	94.7	1.6
37	Chrysin	12.99	C_15_H_10_O_4_	255.065	255.0660	3.2	84.7	2.1
38	Nomilin	13.07	C_28_H_34_O_9_	515.228	515.2286	2.0	89.6	0.2
39	Tangeretin	13.62	C_20_H_20_O_7_	373.128	373.1280	-0.5	98.3	1.6
40	Curdione	13.65	C_15_H_24_O_2_	237.185	237.1853	1.8	92.1	2.0
41	Obacunone	13.67	C_26_H_30_O_7_	455.206	455.2075	2.3	76.3	0.4
42	Costunolide	15.09	C_15_H_20_O_2_	233.154	233.1542	2.5	91.1	1.5
43	*α*-Cyperone	15.41	C_15_H_22_O	219.174	219.1748	1.9	87.8	1.3
1	L(+)-Arginine	1.06	C_6_H_14_N_4_O_2_	173.104	173.1043	-0.6	95.9	0.7
2	Aspartic acid	1.09	C_4_H_7_NO_4_	132.030	132.0302	-0.5	84.0	0.8
3	D-(+)-Glucose	1.18	C_6_H_12_O_6_	179.056	179.0559	-0.9	78.3	0.1
4	Quinic acid	1.23	C_7_H_12_O_6_	191.056	191.0559	-1.1	91.3	1.1
5	Maltopentaose	1.27	C_30_H_52_O_26_	827.267	827.2664	-1.3	97.4	1.6
6	L-Malic acid	1.30	C_4_H_6_O_5_	133.014	133.0141	-1.0	86.4	0.2
7	Citric acid	1.98	C_6_H_8_O_7_	191.020	191.0196	-0.4	99.4	0.3
8	Uridine	2.52	C_9_H_12_N_2_O_6_	243.062	243.0621	-0.8	78.5	1.2
9	Guanosine	4.10	C_10_H_13_N_5_O_5_	282.084	282.0841	-0.9	98.2	2.1
10	Phenprobamate	4.77	C_9_H_11_NO_2_	164.072	164.0714	-1.7	98.4	1.7
11	Protocatechuic acid	5.23	C_7_H_6_O_4_	153.019	153.0191	-1.7	96.0	0.8
12	Vanillic acid	5.52	C_8_H_8_O_4_	167.035	167.0348	-1.3	81.2	2.3
13	L-Tryptophan	5.82	C_11_H_12_N_2_O_2_	203.083	203.0823	-1.7	93.1	0.6
14	Protocatechuic aldehyde	6.14	C_7_H_6_O_3_	137.024	137.0243	-0.7	97.2	0.3
15	Chlorogenic acid	6.23	C_16_H_18_O_9_	353.088	353.0876	-0.5	100.0	0.4
16	A3-N-butyl-4,5-Dihydrophthalide	6.53	C_12_H_16_O_2_	191.108	191.1076	-0.6	77.2	3.3
17	Caffeic acid	6.72	C_9_H_8_O_4_	179.035	179.0349	-0.5	83.5	1.6
18	Rutin	7.61	C_27_H_30_O_16_	609.146	609.1455	-1.0	96.6	1.1
19	p-Coumaric acid	7.66	C_9_H_8_O_3_	163.040	163.0400	-0.7	99.0	0.3
20	Hyperin	7.85	C_21_H_20_O_12_	463.088	463.0878	-0.9	87.3	4.0
21	Neoeriocitrin	7.88	C_27_H_32_O_15_	595.167	595.1663	-0.9	95.1	0.7
22	Liquiritin	7.93	C_21_H_22_O_9_	417.119	417.1187	-1.0	98.1	1.7
23	Isoferulic acid	8.08	C_10_H_10_O_4_	193.051	193.0505	-0.8	97.6	0.3
24	Naringin	8.47	C_27_H_32_O_14_	579.172	579.1712	-1.2	96.2	2.0
25	Isochlorogenic acid C	8.62	C_25_H_24_O_12_	515.119	515.1190	-1.0	98.5	0.1
26	Baicalin	9.36	C_21_H_18_O_11_	445.078	445.0772	-1.0	99.4	1.2
27	Cistanoside D	9.73	C_31_H_40_O_15_	651.229	651.2284	-1.7	88.6	4.5
28	Isoliquiritigenin	9.87	C_15_H_12_O_4_	255.066	255.0660	-1.0	92.7	0.8
29	Eriodictyol	9.96	C_15_H_12_O_6_	287.056	287.0556	-1.7	98.9	2.9
30	Xanthotoxol	10.12	C_11_H_6_O_4_	201.019	201.0190	-1.6	91.2	0.9
31	Wogonin 7-O-glucuronide	10.44	C_22_H_20_O_11_	459.093	459.0928	-1.1	98.1	0.9
32	Naringenin	10.95	C_15_H_12_O_5_	271.061	271.0610	-0.9	99.4	0.5
33	Hesperetin	11.30	C_16_H_14_O_6_	301.072	301.0715	-1.0	95.7	0.7
34	Glycocholic acid	11.34	C_26_H_43_NO_6_	464.302	464.3010	-1.6	78.0	0.3
35	Baicalein	11.64	C_15_H_10_O_5_	269.046	269.0454	-0.6	94.0	2.4
36	Glycoursodeoxycholic acid	11.88	C_26_H_43_NO_5_	448.307	448.3067	-0.3	95.2	0.8
37	Glycyrrhizic acid	12.03	C_42_H_62_O_16_	821.397	821.3955	-1.2	92.1	2.3
38	Eupatilin	12.45	C_18_H_16_O_7_	343.082	343.0819	-1.1	98.1	3.2
39	Chrysin	12.99	C_15_H_10_O_4_	253.051	253.0503	-1.4	78.2	1.2
40	Chrysosplenetin B	13.05	C_19_H_18_O_8_	373.093	373.0924	-1.3	94.3	0.9
41	Ursodeoxycholic acid	13.19	C_24_H_40_O_4_	391.285	391.2848	-1.4	96.4	2.3
42	Asiatic acid	13.77	C_30_H_48_O_5_	487.343	487.3419	-2.1	73.2	2.9
43	Dioscin	14.97	C_45_H_72_O_16_COOH^−^	913.480	913.4789	-1.5	76.9	2.2

**Table 2:** Effect of CFD on PCOS rat ovary weights and diameter and organ index (*n* = 10).

	Weight (g)	Weight of left ovary (mg)	Weight of right ovary (mg)	Ovary organ index	Diameter of left ovary (mm)	Diameter of left ovary (mm)
Control	312.52 ± 17.14	69.2 ± 8.66	70.02 ± 7.89	44.72 ± 5.14	5.89 ± 0.68	6.0 ± 0.68
PCOS	393.74 ± 18.1^▲▲^	157.09 ± 17.61^▲▲^	162.1 ± 19.9^▲▲^	81.06 ± 7.31^▲▲^	7.87 ± 0.75^▲▲^	7.86 ± 0.64^▲▲^
PCOS+L-CFD	383.25 ± 18.36	139.55 ± 11.99^★^	145.76 ± 15.89	74.65 ± 6.11^★^	7.52 ± 0.63	7.58 ± 0.33
PCOS+H-CFD	366.73 ± 12.06^★^	117.5 ± 26.96^★★^	122.8 ± 19.52^★^	65.67 ± 9.4^★^	7.12 ± 0.67^★^	7.05 ± 0.72^★^
PCOS+MH	331.81 ± 11.14^★★^	94.98 ± 19.27^★★^	97.78 ± 8.52^★★^	58.23 ± 8.18^★★^	6.43 ± 0.73^★★^	6.69 ± 0.91^★★^

Compared to the control group, ^▲^*P* < 0.05 and ^▲▲^*P* < 0.01; compared to the PCOS group, ^★^*P* < 0.05 and ^★★^*P* < 0.01.

## Data Availability

The data used to support the findings of this study are included in this manuscript.
